# Reflections on the 25th Congress of the European Society for Pediatric Urology Part 1

**DOI:** 10.3389/fped.2014.00064

**Published:** 2014-06-17

**Authors:** Ricardo González, Pedro Lopez Pereira, Barbara Magda Ludwikowski, Alexander Springer, Raimund Stein, Stephanie Anastasia Warne

**Affiliations:** ^1^Auf der Bult Kinderkrankenhaus, Hannover, Germany; ^2^University Hospital La Paz, Madrid, Spain; ^3^Department of Pediatric Surgery, Medical University Vienna, Vienna, Austria; ^4^Department of Urology, University of Mainz, Mainz, Germany; ^5^Oxford Children’s Hospital, Oxford, UK

**Keywords:** ESPU, hypospadias, myelomeningocele, bladder exstrophy, continent diversion

The 25th annual meeting of the European Society for Pediatric Urology (ESPU) took place in Innsbruck, Austria from the 7th to 10th of May 2014. The Specialty Chief Editor and nine Associate editors of Frontiers in Pediatrics, Pediatric Urology were in attendance. Each was asked to select the two presentations that they considered most significant. Here, we highlight some of the papers that we found of particular interest and that may impact our practices. This report reflects personal preferences of the authors and does not pretend to be comprehensive or to rank the quality of the presentations. We are aware that some important presentations may not be included.

Our selections are presented in the approximate chronological order of presentation at the meeting.

## Session 3: Imaging

The group from Linz, Austria, compared the radiation provided by direct isotopic cystography versus conventional fluoroscopic cystography in children undergoing studies to detect vesicoureteral reflux. Contrary to conventional wisdom, estimated radiation exposure from fluoroscopic cystography was 10 times lower than isotopic cystography. This is an increasingly important issue in pediatrics. It is interesting, that the radiation burden of isotopic studies is higher than our gold standard. Of course, these conclusions may not apply to every imaging department since the radiation exposure is dependent on equipment and protocols used.

## Session 5: Exstrophy–Epispadias Complex

The group from Regensburg, Germany, presented their initial experience with delayed primary closure of classical bladder exstrophy in 28 children. Even when the patients were seen as newborns the operation was done electively at a mean of 66 days of age (44–173). The operation consisted of a complete closure of the bladder, abdominal wall, and epispadias. All patients had an indwelling epidural catheter and no patient had pelvic osteotomies. The pubic diastasis was approximated with PDS sutures in all cases. All patients were successfully closed. Although we will need to wait several years to know the long-term results of this approach, this series negates the dogma that if the closure is postponed beyond 72 h after birth, pelvic osteotomies are necessary for a successful closure. The advantages of this approach compared to the repair done immediately after birth is that it avoids some of the potential complications of neonatal anesthesia, allow the parents time to get sufficient information about the condition of their newborn baby and facilitate the transfer of the patient to a center with expertise in these operations. This paper challenged some dogmas demonstrating that delayed closure may be safer than closure within 48 h and that osteotomies are not necessary even for a delayed closure.

Another presentation in this session analyzed the long follow-up of genetic males with cloacal exstrophy raised as females compared to males with classical bladder exstrophy. There is little published on whether the gender assignment in these patients was actually appropriate in the long term. Researchers from Oklahoma City, USA, evaluated 25 XY patients with cloacal exstrophy who were raised as female and, on reaching adolescence, reverted to male gender. A comparison group consisted of 30 boys with classical bladder exstrophy. The study demonstrates that penis size is not a good criterion for gender assignment in this population and that sexual relations as well as quality of life tend to improve during adulthood. Clinicians are advised to screen for depression/suicidal ideation and to provide psychological support to all patients with these malformations.

## Session 7: Augmentation and Diversion

The group from Indianapolis, USA retrospectively looked at the complication rate of appendicovesicostomy, and catheterizable channels constructed with two techniques of reconfigured ileum in more than 500 patients with a follow-up of more than 5 years.

Fourteen patients with appendicovesicostomy (6.7%) had subfascial revision compared to 47 reconfigured ileal segments with the Yang–Monti technique (15.9%; *p* = 0.002). On multivariate regression Yang–Monti channels were 2.2 times more likely than appendicovesicostomy to require subfascial revision (*p* = 0.03). The spiral reconfiguration of the ileum (Casale) brought out to the umbilicus was five times more likely than the appendicovesicostomy to undergo revision. Gender, age at surgery, and stoma location were not predictors of subfascial revision (*p* > 0.29). This paper demonstrates that the long-term complications of the reconfigured ileum are greater than with the use of the appendix. We all sensed that but this clearly and honest report confirms the feeling.

## Session 9: Testis

We found four presentations here that are worth commenting on.

The first one from Copenhagen, Denmark, reported that serum inhibin-B levels before and after orchidopexy and testicular biopsy in 32 boys (mean age 14 months) with unilateral cryptorchidism. Serum inhibin-B levels raised significantly in 10 patients. All of them had low germ cell numbers in the biopsy. FSH levels were elevated in seven patients before surgery and become normal in six after orchidopexy. Although the practical significance of these findings is elusive, it clearly demonstrates that testicular function may normalize in some boys after orchidopexy. Even though orchidopexy is one of the most frequently performed operations in children, few benefits other than cosmetic and a reduction in testicular malignancies have been shown to date.

Another paper of interest in this session came from a group in Muenster, Germany.

They retrospectively analyzed the records of eight patients aged 14–36 years old with azoospermia and untreated cryptorchidism who underwent testicular sperm extraction. Contrary to expectations, in three patients sperm could be retrieved, in two of poor quality but suitable for cryopreservation and in one case (30-year-old) normal. The authors concluded that in cases of untreated testicular malposition orchiectomy should be postponed until after puberty to have the chance of performing testicular sperm extraction.

A third paper in this session addressed the reliability of preoperative scrotal examination on the ultimate findings in the evaluation of unilateral non-palpable testis. Some have recently proposed that if a “nubbin” is palpated in the scrotum, children with non-palpable testis can be operated through a scrotal approach and laparoscopy (the standard of care) can be avoided. Researchers from Oklahoma City, USA, reported that in their experience with 71 patients, the chance of missing an intra-abdominal testis was 17% if laparoscopy was not performed in patients with non-palpable testes.

Finally, in this session a group from Hamilton, Canada, reported on the inaccuracy of ultrasound to diagnose undescended testes. Although this fact is well known to surgeons, it may be worth reminding our pediatric colleagues that a good physical examination is better.

## Sessions 11 and 12: Hypospadias

A presentation entitled “Penile size and correlation with sexuality in men with corrected hypospadias” from the group at the Children’s Hospital of the University of Zurich, Switzerland, evaluated penile length and sexual function of men with corrected hypospadias and controls consisting of men circumcised at a comparable age. Flaccid and erect penile length in patients with corrected distal hypospadias was similar to controls whereas those with a history of proximal hypospadias repair had somewhat shorter penises. Penile length did not correlate with sexual satisfaction. There are many papers analyzing grade of satisfaction in the adulthood in hypospadias patients who underwent penile reconstruction in childhood but very few analyze penis size and its correlation with sexual function using controls.

Another report on the topic of hypospadias addressed the use of preoperative testosterone on the complication rate of hypospadias repair was presented by the group in Utrecht, Netherlands. When faced with a severely undervirilized male with severe hypospadias many surgeons often give testosterone before the surgery. The goal is to increase penile length, the diameter of the glans, and improve the vascularization of the skin. Recently, some authors have expressed concerns about the effects of testosterone in wound healing and increased complication rate. The retrospective study in question did not find any adverse effect on complication rate, final penile length of adult height of the patients in the study. Prospective studies are needed to determine if preoperative androgen stimulation helps or hinders hypospadias surgery.

The group from Ghent, Belgium, retrospectively analyzed factors leading to re-intervention in a series of 474 operations for hypospadias (77% distal) with a mean follow-up of 23 months. The reoperation rate was 24.1%. They looked at variables such as technique, suture material, and severity of the malformation. The only significant predictor of reoperation was a diagnosis of proximal hypospadias. This study with robust numbers and statistical analysis confirms what experienced surgeons already know, the more proximal the hypospadias the more frequent the need to re-operate.

A group in Glasgow, UK, measured anoscrotal (AS) and anogenital (AG) distance (an expression of fetal androgen exposure during the masculinization programing window) and found that AS and AG distances were shorter in boys with severe hypospadias suggesting reduced androgen exposure during a critical period of development. This is a promising tool that should be applied prospectively.

## Session 22: Neurogenic Bladder

Two presentations in this session reported on result of urodynamic studies in children with *in utero* repaired myelomeningocele. A series from São Paulo, Brazil, included 25 patients with lesions repaired at 25.6 weeks of gestation and delivered at 32.1 weeks. Urodynamic studies were performed between 1 and 24 months of age. There was a variety of urodynamic abnormalities in these children, the most prevalent of were which bladder overactivity (88%) and small bladder capacity (64%). Twenty percent of the patients had vesicoureteral reflux and 40% detrusor–sphincter dyssynergy. It was felt that 52% of the patients required intermittent catheterization and anticholinergics.

In contrast, the group from Zurich, Switzerland compared six children with *in utero* repaired myelomeningocele since 2013 with six children with similar lesions born before 2013 and closed postnatally. Bladder capacity, detrusor pressure, presence of overactivity, and post-void residuum were evaluated at a mean of 20 days and 4.5 months after birth. At the second follow-up children with prenatal repair had lower bladder capacity, detrusor pressure, and residual urine than those closed postnatally. The authors interpreted these findings as being normal in 4/6 children in the *in utero* closed group compared to 0/6 in the postnatally closed group. Clearly larger numbers and longer follow-up are needed to determine the potential urodynamic benefits of *in utero* closure of myelomeningocele.

## Conclusion

We present a subjective report of our experience at the 25th annual meeting of the ESPU. Some of the invited lectures were interesting as was the discussion in most sessions. The meeting was well organized and well attended and we returned home with new ideas for research and to apply to our practice.

## Conflict of Interest Statement

The authors declare that the research was conducted in the absence of any commercial or financial relationships that could be construed as a potential conflict of interest.

